# Correction: Phosphoglycerate mutase 1 promotes cancer cell migration independent of its metabolic activity

**DOI:** 10.1038/s41388-019-1148-0

**Published:** 2020-01-02

**Authors:** D. Zhang, N. Jin, W. Sun, X. Li, B. Liu, Z. Xie, J. Qu, J. Xu, X. Yang, Y. Su, S. Tang, H. Han, D. Chen, J. Ding, M. Tan, M. Huang, M. Geng

**Affiliations:** 1 Division of Antitumor Pharmacology, State Key Laboratory of Drug Research, Shanghai Institute of Materia Medica, Chinese Academy of Sciences, Shanghai, China; 2 The Chemical Proteomics Center, State Key Laboratory of Drug Research, Shanghai Institute of Materia Medica, Chinese Academy of Sciences, Shanghai, China; 3 Laboratory of Pharmaceutical Analysis, Shanghai Institute of Materia Medica, Chinese Academy of Sciences, Shanghai, China

**Correction to: Oncogene**

10.1038/onc.2016.446

Following publication of this Article the Authors noted that a blot in Fig. 1c was misplaced and images were inadvertently duplicated in Supplementary Figs. S2 and S3.

**Figure 1:**
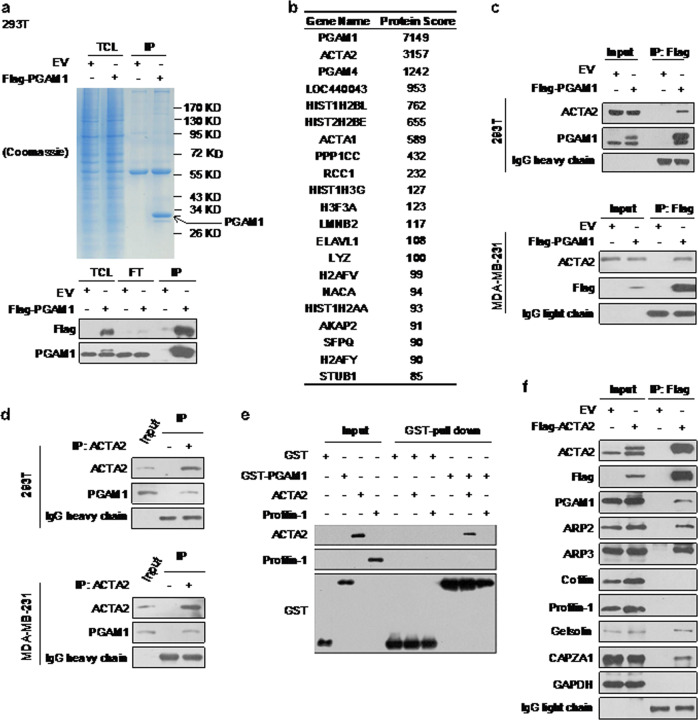
Fig. 1

The corrected Fig. 1 can be found below. The incorrect Supplementary files have been added to this Article. The scientific conclusions of this paper were not affected.

## Supplementary Information

Supplementary Information

## Supplementary Information

**Supplementary information** The online version of this article (10.1038/s41388-019-1148-0) contains supplementary material, which is available to authorized users.

