# Supplementary material for: Correction: Phosphoglycerate mutase 1 promotes cancer cell migration independent of its metabolic activity
Source: Oncogene. 2020 Jan 2;39(11):2451–2. doi: 10.1038/s41388-019-1148-0 (PMC8075972; doi:10.1038/s41388-019-1148-0)
Supplement: Supplementary file 1 — Supplementary Information [file 41388_2019_1148_MOESM1_ESM.docx]

**SUPPLEMENTARY INFORMATION**

Phosphoglycerate Mutase 1 Promotes Cancer Cell Migration Independent of its Metabolic Activity


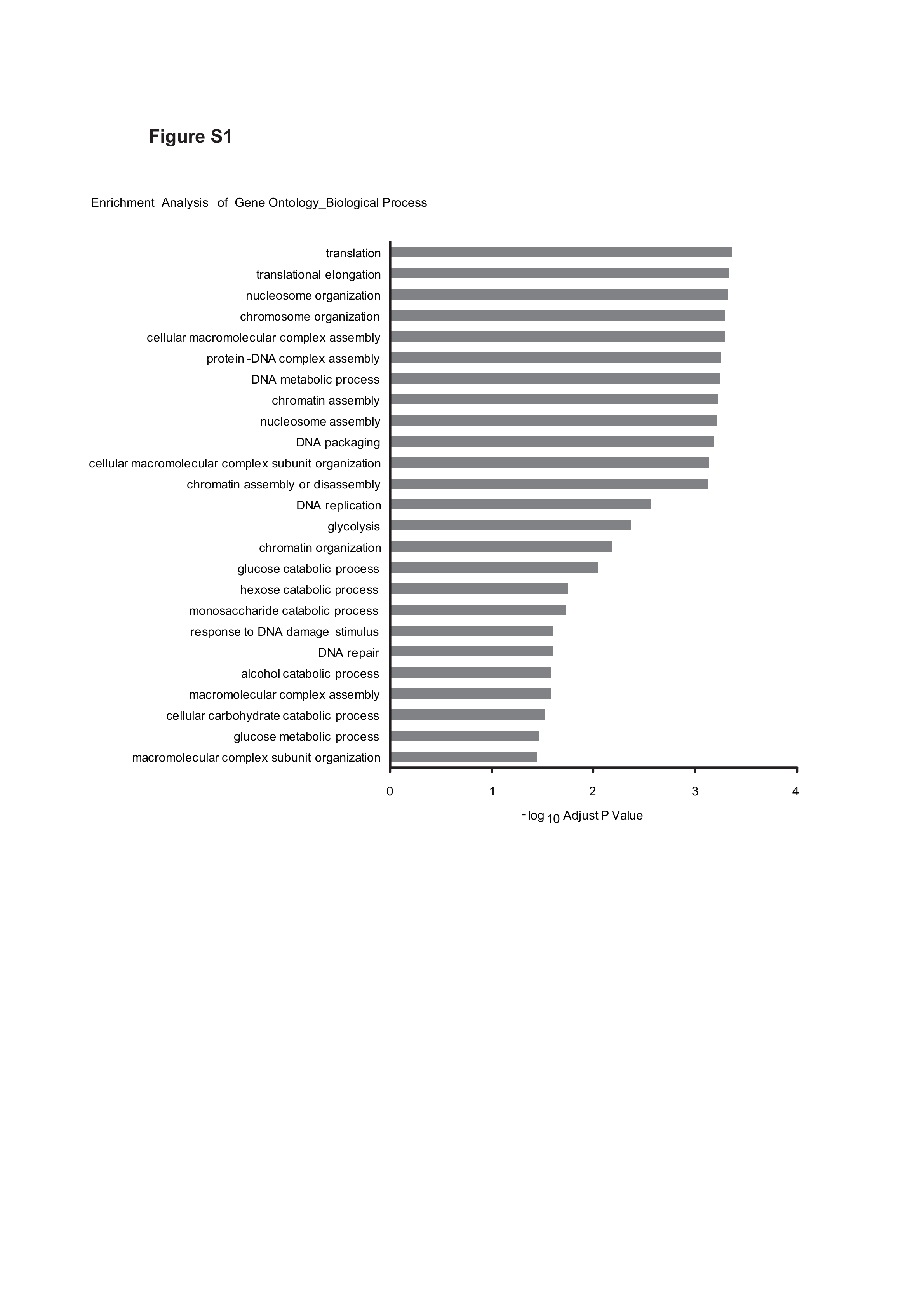


Supplementary figure 1. PGAM1 interacting proteins involved cellular processes.

Enrichment analysis of Gene Oncology/Biological Process on PGAM1 interacting proteins.


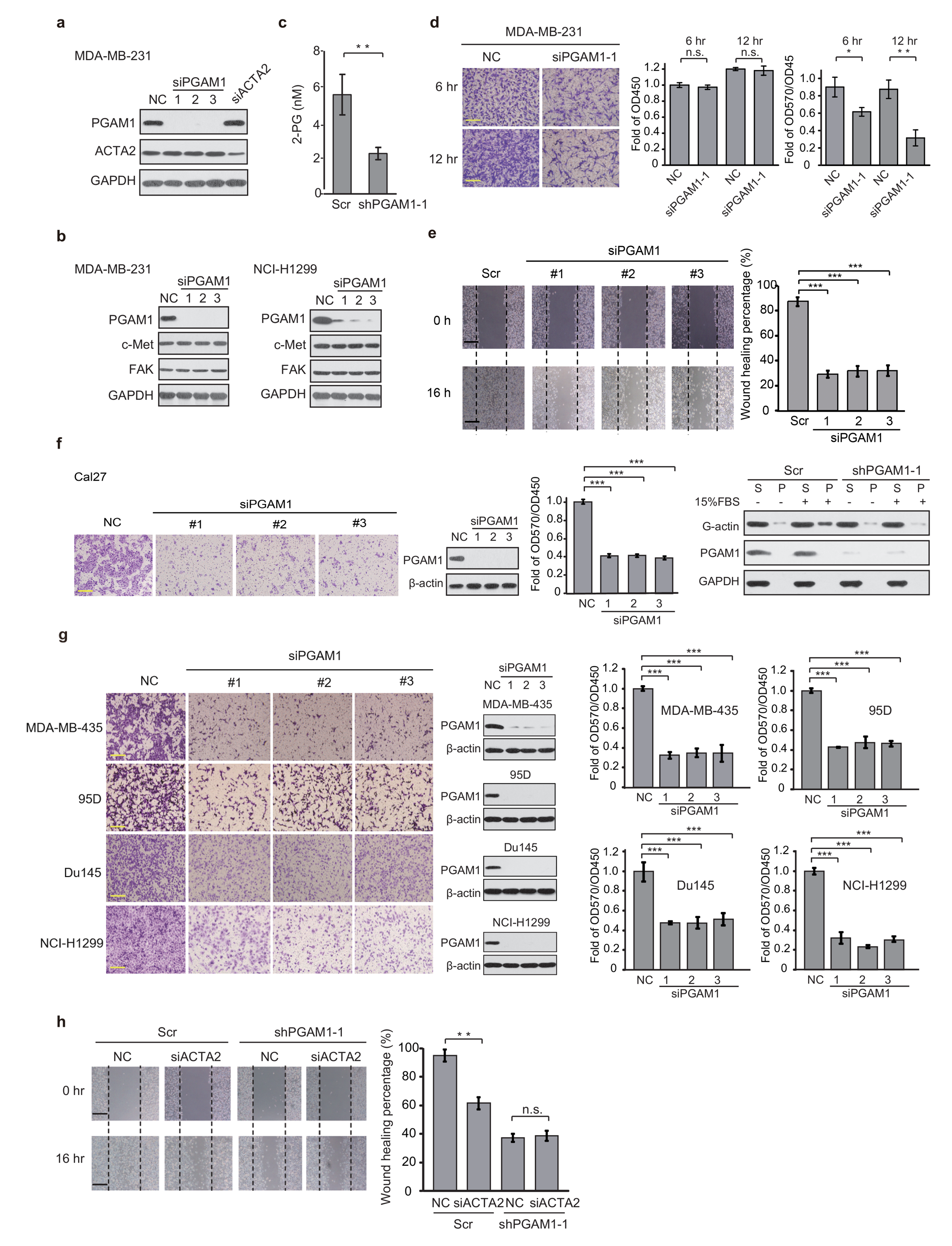
**Supplementary figure 2. PGAM1 promotes actin filaments assembly and cancer cell migration.**

**(a)** The knockdown efficacy of siRNAs targeting PGAM1 or ACTA2 in MDA-MB-231 cells applied to proceeding immunofluorescence assay was measured by immunoblotting; **(b)** The protein levels of c-Met and FAK measured by immunoblotting in MDA-MB-231 cells and NCI-H1299 cells with PGAM1 knockdown; **(c)** Cellular 2-PG level change in PGAM1 stably depleted MDA-MB-231 cells; **(d)** Representative images from transwell migration assay for 6 hr and 12 hr respectively; Cells migrated to the lower chamber were quantified by absorbance measurement (OD570) and were normalized by total cell number (OD450); Bar, 0.25 µm; **(e)** Cell migration change measured by wound-healing assay (left panel) and corresponding quantification (right panel) in PGAM1 depleted MDA-MB-231 cells; Bar, 0.5 µm; **(f)** Cell migration and F-actin assembly alteration in PGAM1 depleted Cal-27 cells; Bar, 0.25 µm; **(g)** Cell migration change measured by transwell assay and corresponding knockdown efficacy in four different cancer cells with PGAM1 depletion. Cells migrated to the lower chamber were quantified as described in (d); Bar, 0.25 µm; **(h)** Cell migration of PGAM1 and ACTA2 concurrently depleted MDA-MB-231 cells measured by wound-healing assay (left panel) and corresponding quantification (right panel); Bar, 0.5 µm. The error bars represent mean values ± SD from three independent experiments; Two tail student-t test analysis was used to compare the statistic difference between indicated two groups in (c), (d) and (h); One-way ANOVA followed by Dunnett's post hoc test was applied in experiments containing multiple groups in (e), (f) and (g); ^*^p < 0.05; ^**^p < 0.01; ^***^p < 0.001; n.s., not significant. NC, negative control.


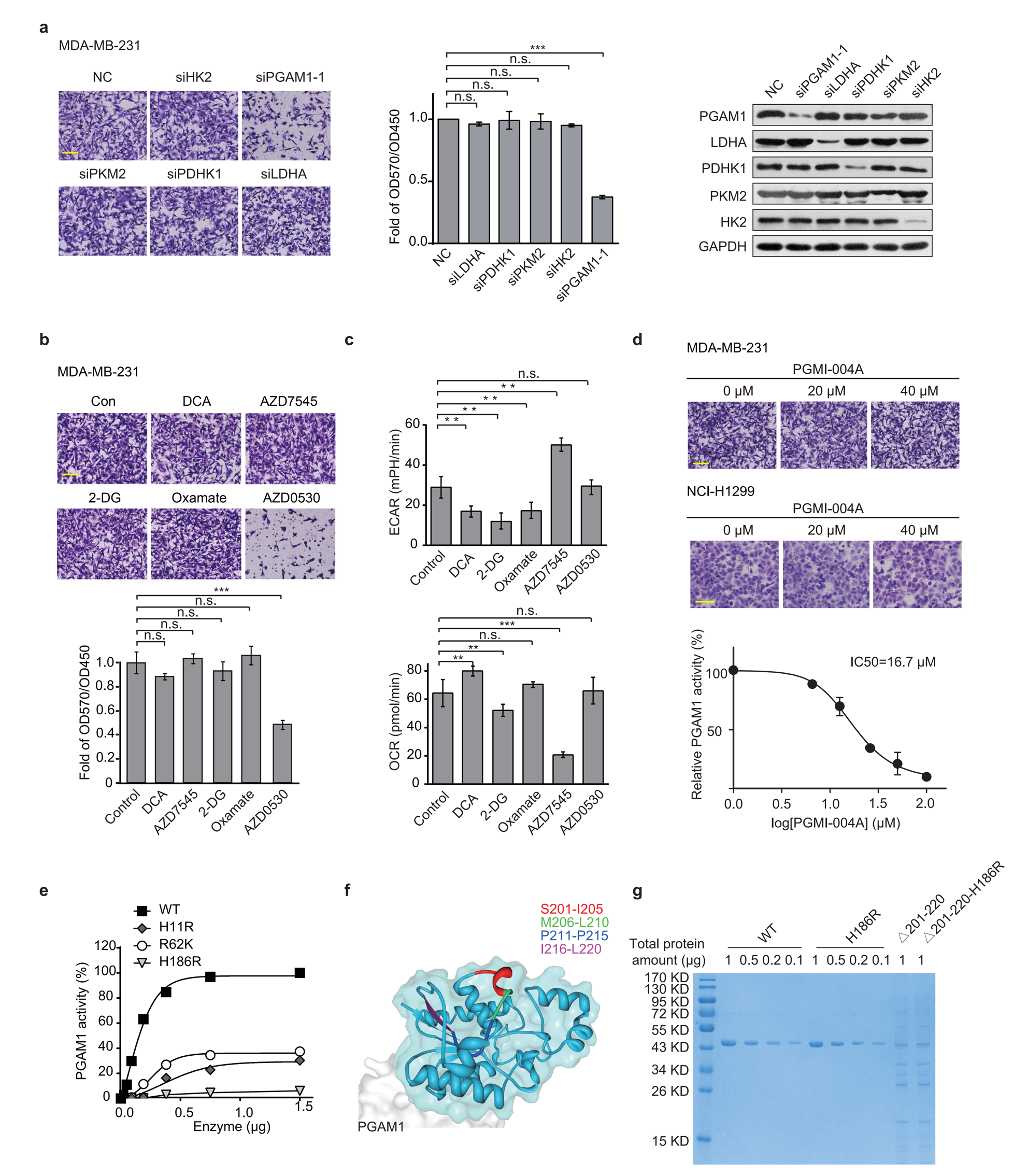


**
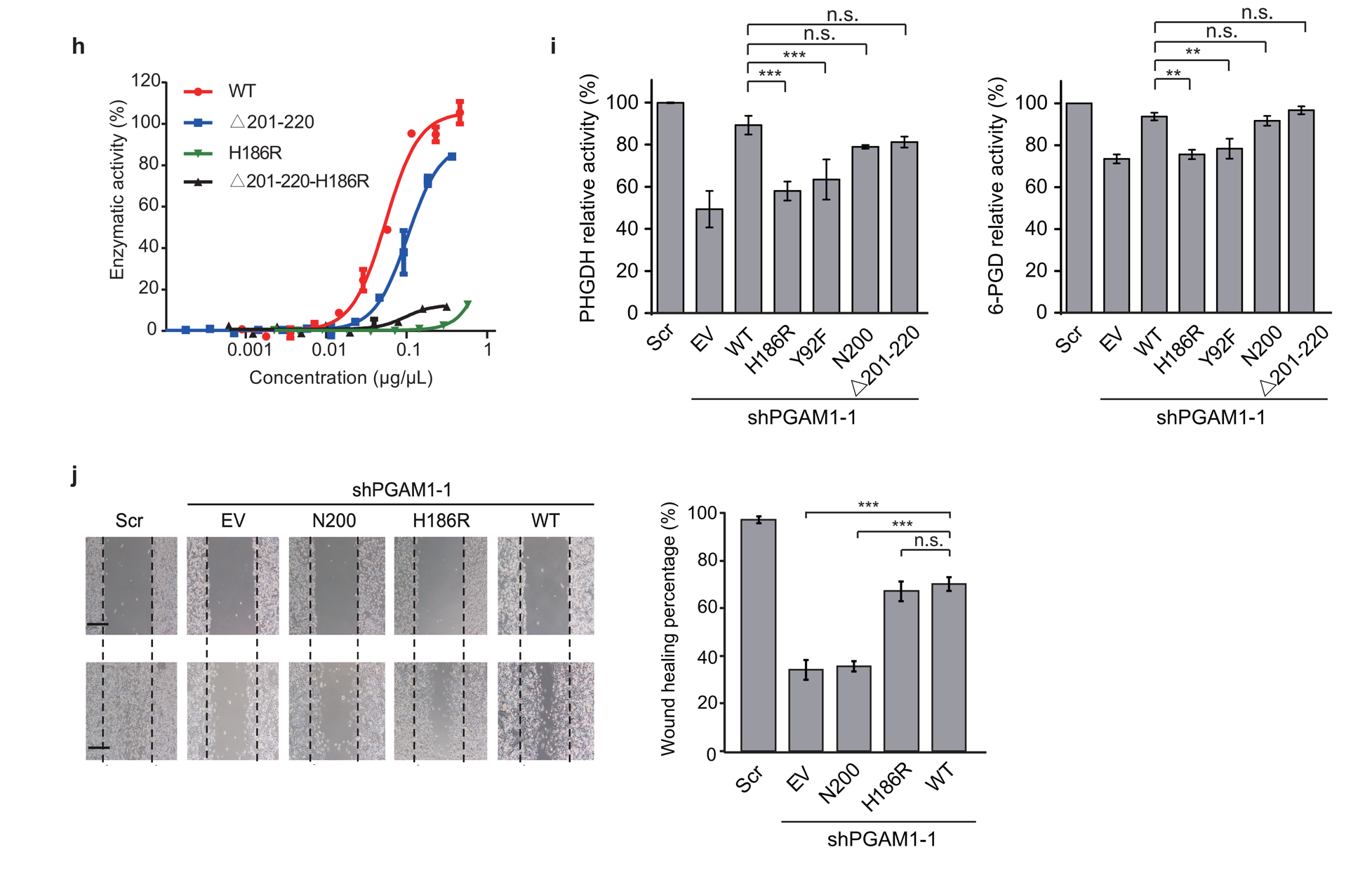
**

**Supplementary figure 3. PGAM1 promotes cell migration independent of its metabolic activity**

**(a)** Cell migration measured by transwell assay of cells transfected with indicated siRNAs, and the knockdown efficacy of siRNAs were determined by western blotting; Bar, 0.25 µm; **(b)** Cell migration measured by transwell assay after the treatment with indicated inhibitors in MDA-MB-231 cells for 24 hr; DCA, 10 mM; AZD7545, 10 μM; 2-DG, 20 mM; Oxamate, 20μM. Representative images and quantification were shown; Bar, 0.25 µm; **(c)** ECAR and OCR measured by seahorse XF after treated with indicated inhibitors as (b) in MDA-MB-231 cells; **(d)** Cell migration measured by transwell assay after treated with PGAM1-004A at indicated concentrations for 12 hr in MDA-MB-231 cells and NCI-H1299 cells (up panel); PGAM1 activity inhibited by PGMI-004A (down panel); Bar, 0.25 µm; **(e)** Protein titration of PGAM1 mutants in the enzymatic assay; (**f**) The model of 3-dimentional crystal structure of PGAM1 (PDB: 4GPZ), and amino acid residues between 201-210 were distinguished by different colors; (**g**) Coomassie blue staining of purified PGAM1 wild-type (WT) and indicated mutants; Protein amounts indicate the total protein elution resulted from purification. WT and H186R mutant were loaded in serial dilution for quantitative analysis; (**h**) Enzymatic kinetics of indicated PGAM1 mutant. The protein amount of Δ201-220 and Δ201-220-H186R was quantified according to (g) to exclude the interference of untargeted bands; **(i)** Intracellular PHGDH activity (left panel) and 6-PGD activity (right panel) in PGAM1 stably depleted MDA-MB-231 cells reconstituted with PGAM1 wild type or indicated mutants; **(j)** Cell migration measured by wound-healing assay in PGAM1 depleted MDA-MB-231 cells reconstituted with PGAM1 wild type or indicated mutants (left panel) and corresponding quantification (right panel); Bar, 0.5 µm. The error bars represent mean values ± SD from three replicates. One-way ANOVA followed by Dunnett's post hoc test was used for statistical analysis. ^**^p <0.01; ^***^p <0.001; n.s., not significant; NC, negative control; Scr, negative control vector containing scrambled shRNA; EV, empty vector; WT, wild type.


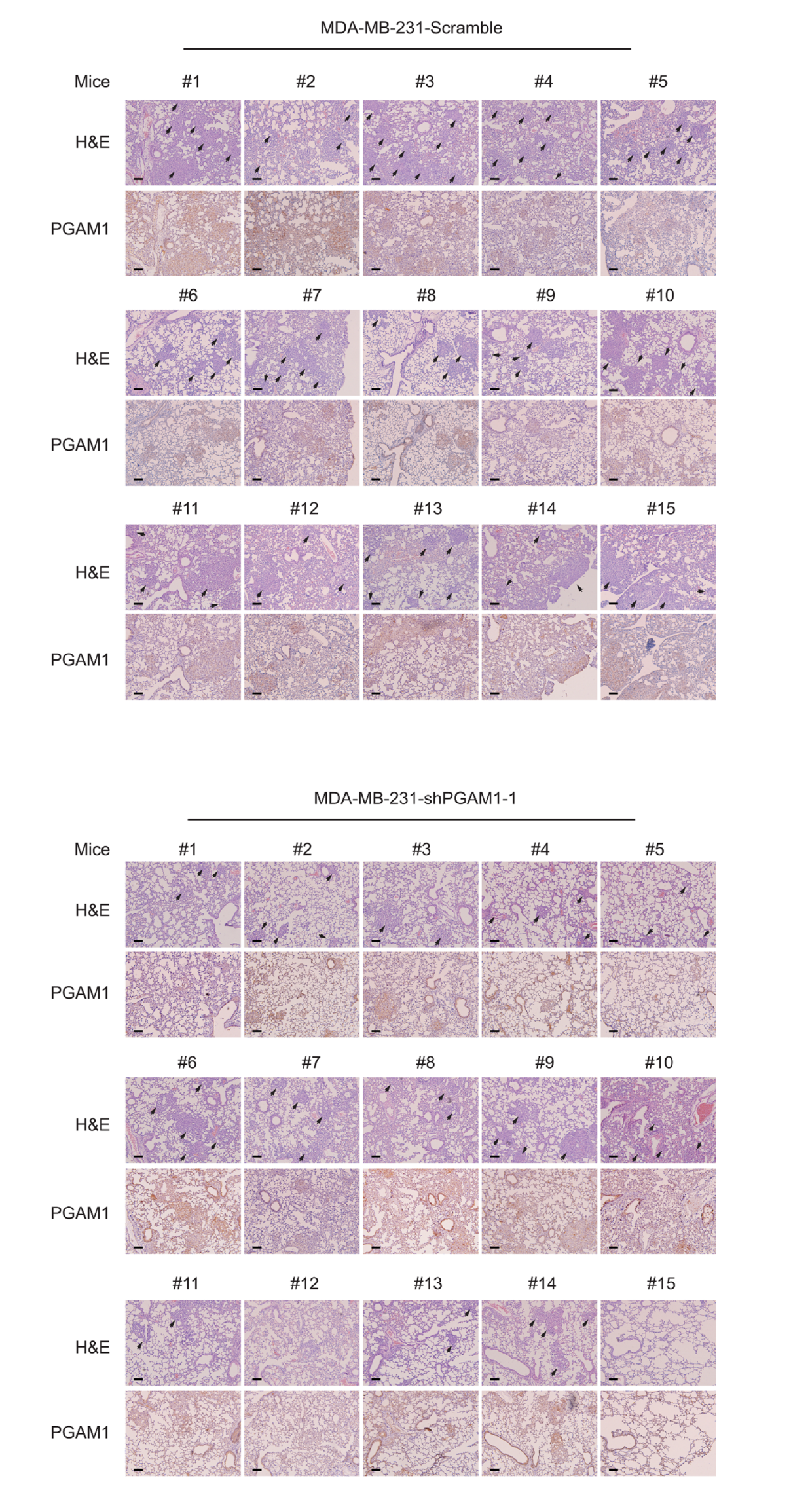


**Supplementary figure 4. Depletion of PGAM1 decreases tumor metastasis in vivo.**

Representative images of H&E staining or PGAM1 staining by immunohistochemistry of mice lung section from experimental metastasis study using PGAM1 stably depleted cells or scrambled control cells (15 mice per group). Shown are representative field from one section per mouse; Arrows indicates the metastatic foci; # indicates individual mice identity in each group; Bar, 0.1 µm.


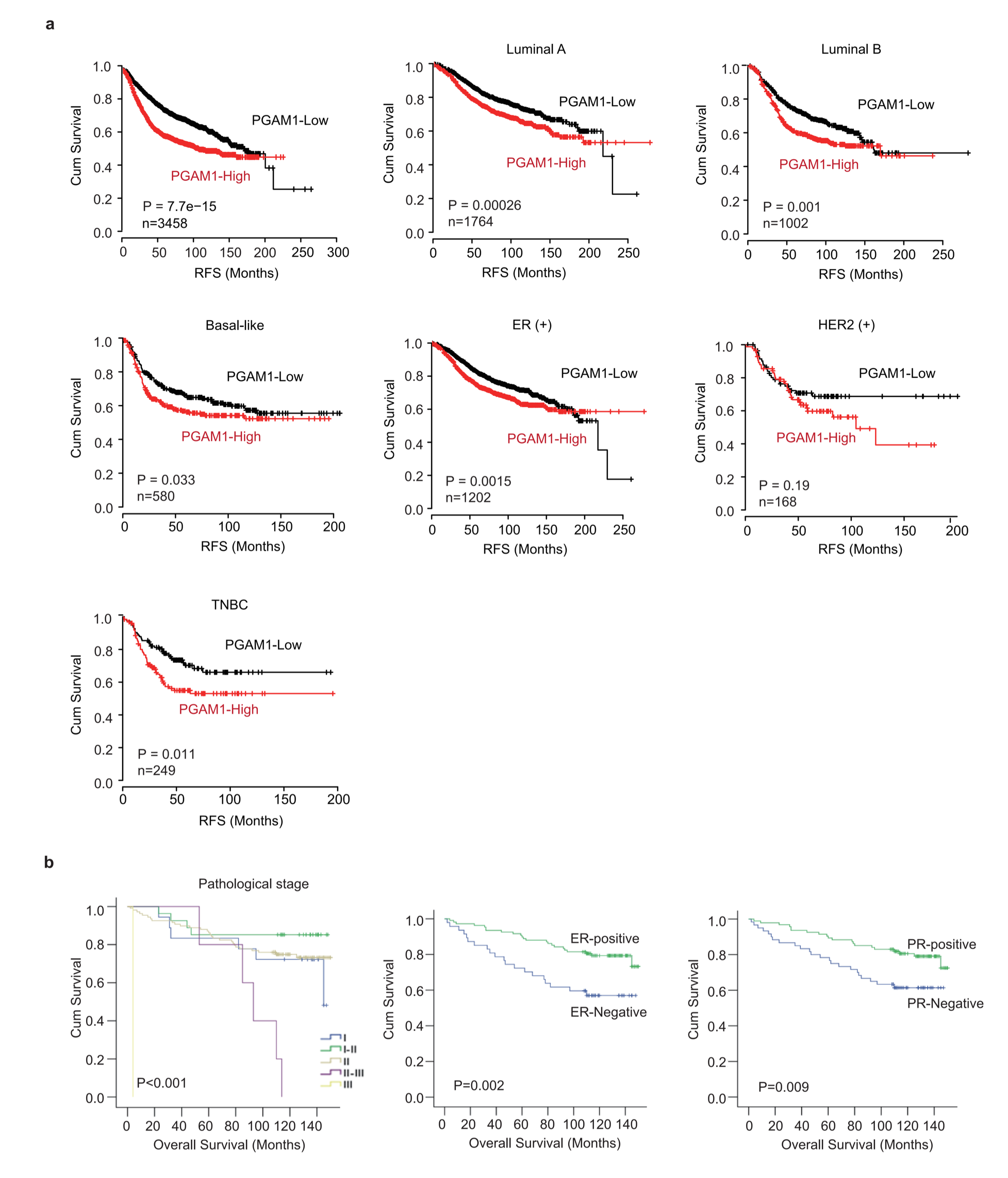


**Supplementary figure 5. Association between PAGM1 expression, pathological stage, estrogen receptor (ER), or progesterone receptor (PR) status with prognosis of breast cancer patients.**

**(a)** Correlation between PGAM1 expression with RFS of breast cancer patients by Kaplan-Meier survival analysis in a breast cancer patient cohort and indicated subtypes; **(b)** Correlation between pathological stage, ER or PR status with overall survival of breast cancer patients (n = 160). Log-rank test was used to analyze the difference between the groups.

**
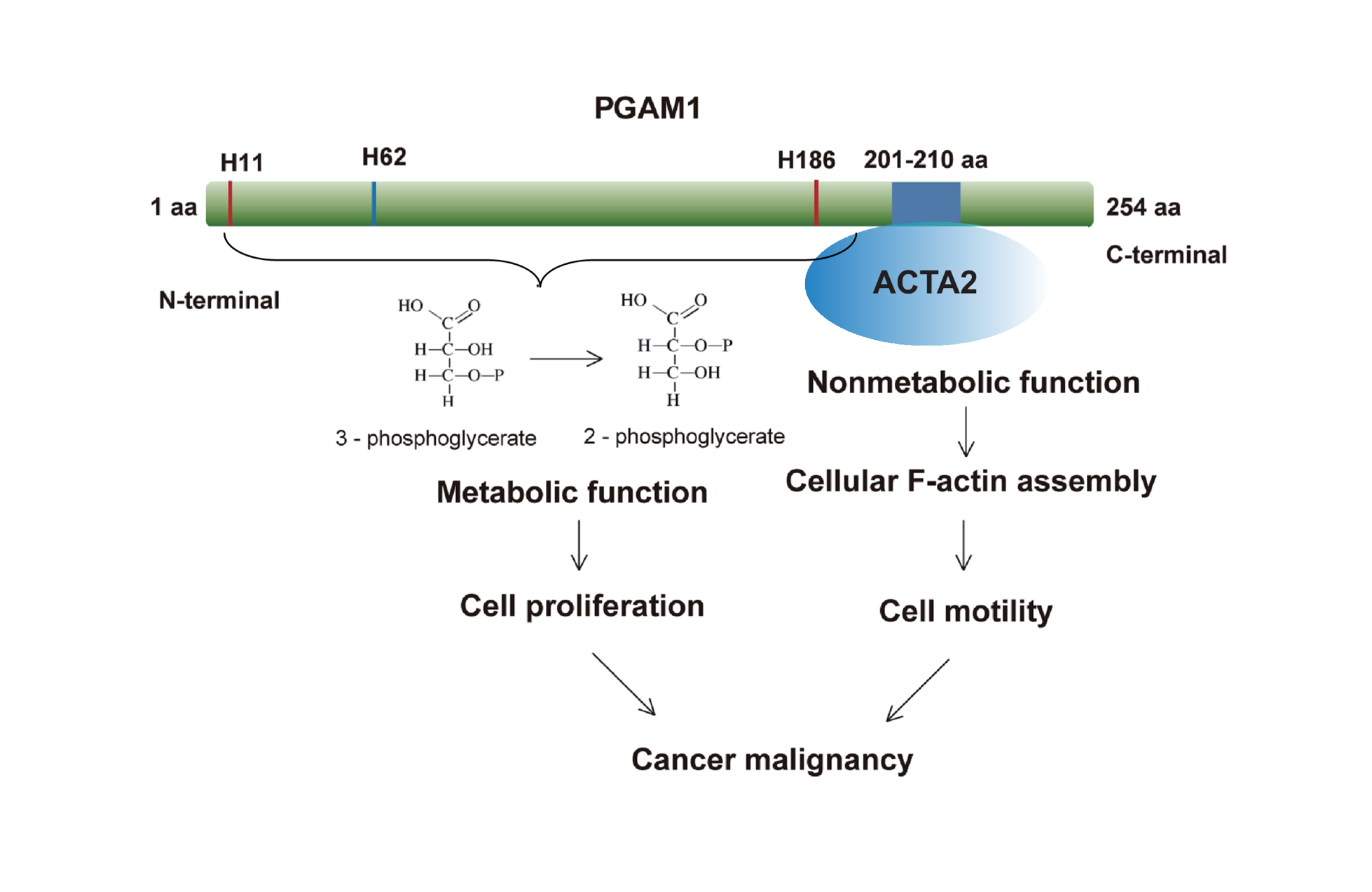
**

**Supplementary figure 6. A proposed model demonstrating the metabolic and non-metabolic role of PGAM1 in cancer malignancy.**

Supplementary Table 1. The list of PGAM1 interacting proteins.

| **Gene Name** | **Protein Score** | **Gene Name** | **Protein Score** |
| --- | --- | --- | --- |
| PGAM1 | 7149 | RPL9 | 52 |
| ACTA2 | 3157 | RPL29 | 52 |
| PGAM4 | 1242 | XRCC1 | 47 |
| LOC440043 | 953 | POLB | 45 |
| HIST1H2BL | 762 | C19orf21 | 42 |
| HIST2H2BE | 655 | RAN | 41 |
| ACTA1 | 589 | ANXA2P2 | 41 |
| PPP1CC | 432 | RPS10 | 39 |
| RCC1 | 232 | RPL26L1 | 39 |
| HIST1H3G | 127 | RPA1 | 39 |
| H3F3A | 123 | PES1 | 39 |
| LMNB2 | 117 | SNRPB | 38 |
| ELAVL1 | 108 | MED24 | 37 |
| LYZ | 100 | RPS24 | 36 |
| H2AFV | 99 | PKM2 | 36 |
| NACA | 94 | PAICS | 36 |
| HIST1H2AA | 93 | DRD3 | 36 |
| AKAP2 | 91 | C17orf79 | 36 |
| SFPQ | 90 | SVIL | 35 |
| H2AFY | 90 | RPL35A | 35 |
| STUB1 | 85 | LOC653479 | 35 |
| SSRP1 | 83 | IGF2BP3 | 35 |
| PRKDC | 83 | TUBA1C | 34 |
| TPM1 | 81 | TOP2A | 34 |
| RPL10A | 81 | LOC100290864 | 34 |
| LIG3 | 79 | KIAA0586 | 34 |
| LDHAL6A | 74 | U2AF1 | 33 |
| BPGM | 72 | PRSS1 | 33 |
| HSPD1 | 71 | PCDH17 | 33 |
| COX7A2 | 70 | ATXN10 | 33 |
| HNRNPAB | 68 | SSH2 | 32 |
| SIPA1 | 66 | PEX26 | 32 |
| TXN | 65 | MYBBP1A | 32 |
| EPPK1 | 62 | TOP1 | 31 |
| RPS6 | 59 | TMEM206 | 31 |
| SNRPF | 58 | SBNO2 | 31 |
| IGF2BP1 | 58 | MCM7 | 31 |
| RNH1 | 57 | LRRFIP2 | 31 |
| FHL1 | 55 | EFHC1 | 31 |
| ABCF1 | 55 | TMEM131 | 30 |
| RBBP4 | 54 | NDR1 | 30 |
| HNRNPL | 54 | GNB2 | 30 |
| LCN1 | 53 | CHD9 | 30 |

Proteins were determined by LC-MS and searched against UniProt Mus Musculus database. The identified proteins were listed in the order of protein score. The higher the protein score indicates the higher confidence of the protein identification.

Supplementary Table 2. The information of tissue microarray assay

| **Sample  number** | **State*** | **Lifetime (Month)** | **Pathological grading** | **ER  IHC** | **PR  IHC** | **HER2  IHC** | **PGAM1 IHC** | | | **ACTA2 IHC** | | |
| --- | --- | --- | --- | --- | --- | --- | --- | --- | --- | --- | --- | --- |
|  |  |  |  |  |  |  | **Intensity score** | **Positive  rate (%)** | **Classification** | **Intensity score** | **Positive  rate (%)** | **Classification** |
| J07A0929 | 0 | 150 | Ⅱ | + | + | - | 1 | 70 | Low | 1 | 90 | Medium |
| J07A0930 | 0 | 150 | Ⅱ | + | - | - | 1 | 90 | Medium | 1 | 50 | Low |
| J07A0931 | 0 | 150 | Ⅱ | - | - | - | 1 | 90 | Medium | 1 | 50 | Low |
| J07A0933 | 1 | 32 | Ⅰ-Ⅱ | + | + | - | 1.5 | 90 | High | 1.5 | 90 | High |
| J07A0934 | 0 | 149 | Ⅱ | + | + | - | 1 | 90 | Medium | 1.5 | 90 | High |
| J07A0935 | 0 | 148 | Ⅱ | - | - | - | 1 | 80 | Low | 1 | 90 | Medium |
| J07A0936 | 0 | 148 | Ⅰ-Ⅱ | + | + | - | 1 | 50 | Low | 1 | 50 | Low |
| J07A0937 | 1 | 147 | Ⅰ-Ⅱ | + | + | - | 1 | 10 | Low | 1 | 90 | Medium |
| J07A0938 | 1 | 145 | Ⅰ | + | - | - | 1.5 | 90 | High | 1 | 90 | Medium |
| J07A0939 | 0 | 147 | Ⅱ | - | - | + | 1 | 10 | Low | 1 | 90 | Medium |
| J07A0940 | 0 | 147 | Ⅰ-Ⅱ | + | + | - | 1.5 | 90 | High | 1.5 | 90 | High |
| J07A0941 | 0 | 147 | Ⅰ | + | + | - | 1 | 50 | Low | 1 | 90 | Medium |
| J07A0942 | 1 | 33 | Ⅱ | - | - | - | 1.5 | 90 | High | 1.5 | 90 | High |
| J07A0943 | 0 | 146 | Ⅱ | + | + | - | 1.5 | 90 | High | 1 | 90 | Medium |
| J07A0944 | 0 | 145 | Ⅱ | + | - | - | 1 | 70 | Low | 1 | 90 | Medium |
| J07A0945 | 1 | 54 | Ⅱ | - | - | + | 1 | 80 | Low | 1 | 90 | Medium |
| J07A0946 | 0 | 145 | Ⅰ | - | - | + | 1 | 50 | Low | 1 | 90 | Medium |
| J07A0947 | 1 | 93 | Ⅱ-Ⅲ | - | - | - | 1 | 80 | Low | 1 | 90 | Medium |
| J07A0951 | 0 | 145 | Ⅱ | + | + | - | 1 | 70 | Low | 1 | 80 | Low |
| J07A0952 | 1 | 114 | Ⅱ-Ⅲ | + | + | - | 1.5 | 90 | High | 1 | 90 | Medium |
| J07A0954 | 0 | 145 | Ⅱ | - | - | + | 1 | 90 | Medium | 1 | 90 | Medium |
| J07A0956 | 1 | 97 | Ⅱ | - | - | - | 1 | 80 | Low | 1 | 90 | Medium |
| J07A0957 | 0 | 144 | Ⅱ | + | + | + | 1 | 50 | Low | 1 | 80 | Low |
| J07A0958 | 0 | 143 | Ⅱ | + | + | - | 1 | 90 | Medium | 1 | 90 | Medium |
| J07A0960 | 0 | 143 | Ⅱ | - | - | + | 1 | 90 | Medium | 1 | 90 | Medium |
| J07A0962 | 0 | 143 | Ⅰ-Ⅱ | - | - | + | 1 | 80 | Low | 1 | 90 | Medium |
| J07A0963 | 1 | 18 | Ⅱ | - | - | - | 1.5 | 90 | High | 1.5 | 90 | High |
| J07A0964 | 1 | 17 | Ⅱ | - | - | + | 1.5 | 90 | High | 1.5 | 90 | High |
| J07A0965 | 0 | 143 | Ⅰ-Ⅱ | + | + | - | 1 | 70 | Low | 1 | 90 | Medium |
| J07A0966 | 1 | 11 | Ⅱ | - | - | - | 1.5 | 90 | High | 1.5 | 90 | High |
| J07A0967 | 1 | 95 | Ⅰ | + | + | - | 1.5 | 90 | High | 1.5 | 90 | High |
| J07A0968 | 1 | 82 | Ⅰ | - | - | - | 1.5 | 80 | High | 1.5 | 90 | High |
| J07A0969 | 0 | 142 | Ⅱ | - | - | + | 1 | 90 | Medium | 1 | 90 | Medium |
| J07A0970 | 0 | 142 | Ⅱ | + | + | - | 1 | 80 | Low | 1 | 90 | Medium |
| J07A0973 | 0 | 141 | Ⅰ | + | + | - | 1 | 50 | Low | 1 | 90 | Medium |
| J07A0974 | 0 | 141 | Ⅰ-Ⅱ | + | + | - | 1 | 80 | Low | 1.5 | 90 | High |
| J07A0975 | 0 | 141 | Ⅰ | + | + | - | 0 | 0 | Low | 1 | 10 | Low |
| J07A0976 | 0 | 141 | Ⅰ-Ⅱ | + | + | - | 1.5 | 90 | High | 1.5 | 90 | High |
| J07A0977 | 0 | 141 | Ⅱ | + | + | - | 1 | 90 | Medium | 1 | 90 | Medium |
| J07A0978 | 0 | 141 | Ⅰ-Ⅱ | + | + | + | 1 | 90 | Medium | 1 | 90 | Medium |
| J07A0979 | 1 | 39 | Ⅱ | - | - | - | 1 | 50 | Low | 1.5 | 90 | High |
| J07A0980 | 0 | 140 | Ⅱ | + | + | - | 1.5 | 90 | High | 1 | 90 | Medium |
| J07A0981 | 0 | 140 | Ⅰ-Ⅱ | + | + | - | 1 | 90 | Medium | 1 | 90 | Medium |
| J07A0983 | 0 | 139 | Ⅰ-Ⅱ | - | - | - | 1.5 | 90 | High | 1.5 | 90 | High |
| J07A0985 | 0 | 138 | Ⅱ | + | + | - | 1 | 80 | Low | 1.5 | 90 | High |
| J07A0986 | 0 | 138 | Ⅱ | - | + | - | 1.5 | 90 | High | 1.5 | 90 | High |
| J07A0987 | 1 | 32 | Ⅰ | + | + | - | 1 | 80 | Low | 1 | 90 | Medium |
| J07A0988 | 0 | 138 | Ⅰ | + | + | - | 1 | 80 | Low | 1 | 90 | Medium |
| J07A0989 | 0 | 137 | Ⅰ | + | + | - | 0 | 0 | Low | 1 | 10 | Low |
| J07A0990 | 0 | 137 | Ⅱ | + | + | - | 1 | 70 | Low | 1 | 90 | Medium |
| J07A0991 | 1 | 7 | Ⅱ | - | - | + | 1 | 80 | Low | 1 | 90 | Medium |
| J07A0992 | 0 | 137 | Ⅰ | + | + | + | 1 | 80 | Low | 1 | 90 | Medium |
| J07A0993 | 0 | 137 | Ⅱ | - | - | + | 1 | 90 | Medium | 1 | 90 | Medium |
| J07A0994 | 0 | 137 | Ⅱ | - | + | - | 1 | 90 | Medium | 1 | 90 | Medium |
| J07A0995 | 0 | 137 | Ⅰ-Ⅱ | / | / | / | 1.5 | 70 | High | 1 | 80 | Low |
| J07A0996 | 1 | 83 | Ⅱ | + | - | - | 1 | 90 | Medium | 1 | 90 | Medium |
| J07A0997 | 0 | 136 | Ⅱ | + | + | - | 1 | 90 | Medium | 1.5 | 90 | High |
| J07A0998 | 0 | 136 | Ⅰ | + | + | - | 1 | 70 | Low | 1 | 90 | Medium |
| J07A0999 | 1 | 125 | Ⅱ | + | + | - | 1 | 30 | Low | 1 | 80 | Low |
| J07A1000 | 1 | 23 | Ⅰ | - | - | + | 1.5 | 70 | High | 1 | 90 | Medium |
| J07A1001 | 0 | 135 | Ⅰ-Ⅱ | - | - | - | 1 | 80 | Low | 1 | 90 | Medium |
| J07A1003 | 0 | 135 | Ⅱ | + | + | - | 1 | 80 | Low | 1 | 90 | Medium |
| J07A1004 | 0 | 135 | Ⅰ-Ⅱ | + | + | - | 1 | 80 | Low | 1 | 90 | Medium |
| J07A1006 | 0 | 135 | Ⅰ-Ⅱ | - | - | - | 1 | 90 | Medium | 1 | 90 | Medium |
| J07A1007 | 1 | 47 | Ⅰ-Ⅱ | - | - | - | 1 | 50 | Low | 1 | 90 | Medium |
| J07A1010 | 1 | 77 | Ⅱ | + | - | + | 1 | 90 | Medium | 1 | 90 | Medium |
| J07A1011 | 0 | 134 | Ⅱ | + | + | - | 1 | 70 | Low | 1 | 90 | Medium |
| J07A1012 | 0 | 134 | Ⅱ | + | + | - | 1 | 70 | Low | 1 | 90 | Medium |
| J07A1013 | 1 | 92 | Ⅱ | + | - | - | 1 | 30 | Low | 1 | 90 | Medium |
| J07A1014 | 1 | 31 | Ⅰ | + | + | - | 1 | 10 | Low | 1 | 90 | Medium |
| J07A1017 | 0 | 133 | Ⅱ | + | + | - | 1 | 90 | Medium | 1.5 | 90 | High |
| J07A1018 | 0 | 133 | Ⅰ | + | + | - | 1 | 30 | Low | 1 | 90 | Medium |
| J07A1019 | 0 | 132 | Ⅰ-Ⅱ | - | - | - | 1.5 | 90 | High | 1.5 | 90 | High |
| J07A1020 | 0 | 132 | Ⅱ | + | + | - | 1 | 70 | Low | 1 | 90 | Medium |
| J07A1021 | 0 | 132 | Ⅱ | - | - | - | 1.5 | 90 | High | 1.5 | 90 | High |
| J07A1022 | 0 | 132 | Ⅱ | + | + | - | 1 | 70 | Low | 1.5 | 90 | High |
| J07A1023 | 1 | 53 | Ⅱ-Ⅲ | + | + | - | 2 | 90 | High | 1.5 | 90 | High |
| J07A1024 | 0 | 131 | Ⅱ | + | / | - | 1.5 | 90 | High | 1 | 90 | Medium |
| J07A1026 | 0 | 131 | Ⅱ | + | + | - | 1 | 90 | Medium | 1 | 90 | Medium |
| J07A1028 | 1 | 23 | Ⅰ-Ⅱ | + | + | - | 1 | 70 | Low | 1 | 90 | Medium |
| J07A1029 | 0 | 130 | Ⅱ | + | + | - | 1 | 70 | Low | 1 | 80 | Low |
| J07A1030 | 0 | 130 | Ⅰ-Ⅱ | + | + | - | 1 | 30 | Low | 1 | 80 | Low |
| J07A1031 | 0 | 130 | Ⅱ | + | - | + | 1 | 90 | Medium | 1 | 90 | Medium |
| J07A1032 | 1 | 15 | Ⅱ | - | - | + | 1 | 90 | Medium | 1 | 90 | Medium |
| J07A1033 | 0 | 129 | Ⅰ | + | + | - | 1 | 10 | Low | 1 | 90 | Medium |
| J07A1034 | 1 | 60 | Ⅱ | + | + | - | 1.5 | 90 | High | 1.5 | 90 | High |
| J07A1036 | 1 | 110 | Ⅱ-Ⅲ | + | + | - | 1 | 90 | Medium | 1 | 90 | Medium |
| J07A1037 | 0 | 129 | Ⅰ | + | + | - | 1.5 | 60 | Medium | 1 | 70 | Low |
| J07A1038 | 0 | 128 | Ⅱ | + | + | + | 1 | 80 | Low | 1 | 90 | Medium |
| J07A1039 | 0 | 128 | Ⅱ | + | + | - | 1 | 10 | Low | 1 | 90 | Medium |
| J07A1040 | 1 | 63 | Ⅱ | + | + | + | 1.5 | 90 | High | 1 | 90 | Medium |
| J07A1041 | 1 | 35 | Ⅱ | - | - | - | 1.5 | 90 | High | 1.5 | 90 | High |
| J07A1042 | 0 | 128 | Ⅱ | + | + | - | 1 | 80 | Low | 1 | 90 | Medium |
| J07A1043 | 0 | 128 | Ⅱ | + | + | - | 1 | 70 | Low | 1 | 90 | Medium |
| J07A1044 | 0 | 128 | Ⅱ | + | + | - | 1.5 | 90 | High | 1 | 90 | Medium |
| J07A1045 | 0 | 128 | Ⅱ | + | - | - | 1.5 | 90 | High | 1 | 90 | Medium |
| J07A1046 | 1 | 44 | Ⅰ-Ⅱ | + | + | - | 1 | 90 | Medium | 1 | 90 | Medium |
| J07A1047 | 1 | 78 | Ⅱ | + | + | - | 1 | 50 | Low | 1 | 90 | Medium |
| J07A1049 | 0 | 128 | Ⅱ | + | + | + | 1 | 50 | Low | 1 | 50 | Low |
| J07A1052 | 0 | 127 | Ⅱ | + | + | - | 1 | 70 | Low | 1 | 90 | Medium |
| J07A1053 | 1 | 4 | Ⅱ | - | - | - | 1 | 90 | Medium | 1 | 90 | Medium |
| J07A1054 | 0 | 127 | Ⅰ-Ⅱ | + | + | - | 1.5 | 90 | High | 1.5 | 90 | High |
| J07A1055 | 1 | 4 | Ⅲ | + | + | - | 1 | 90 | Medium | 1 | 90 | Medium |
| J07A1056 | 0 | 127 | Ⅱ | + | - | - | 1 | 80 | Low | 1 | 90 | Medium |
| J07A1057 | 0 | 126 | Ⅱ | + | + | - | 1 | 80 | Low | 1 | 90 | Medium |
| J07A1059 | 1 | 62 | Ⅱ | - | - | - | 1.5 | 90 | High | 1.5 | 90 | High |
| J07A1060 | 0 | 120 | Ⅱ | - | - | + | 1.5 | 90 | High | 1.5 | 90 | High |
| J07A1061 | 0 | 120 | Ⅱ | + | + | - | 1.5 | 90 | High | 1 | 90 | Medium |
| J07A1062 | 0 | 119 | Ⅱ | - | - | + | 1 | 50 | Low | 1 | 50 | Low |
| J07A1063 | 0 | 119 | Ⅱ | - | - | - | 1 | 90 | Medium | 1 | 90 | Medium |
| J07A1067 | 1 | 2 | Ⅱ | - | - | - | 1.5 | 90 | High | 1.5 | 90 | High |
| J07A1068 | 1 | 110 | Ⅱ | - | - | - | 1.5 | 90 | High | 1.5 | 90 | High |
| J07A1070 | 0 | 118 | Ⅱ | + | - | + | 1 | 80 | Low | 1 | 90 | Medium |
| J07A1072 | 0 | 96 | Ⅱ | + | + | - | 1 | 90 | Medium | 1.5 | 90 | High |
| J07A1073 | 0 | 118 | Ⅱ | + | + | - | 1 | 90 | Medium | 1 | 90 | Medium |
| J07A1074 | 0 | 117 | Ⅱ | - | - | - | 1 | 90 | Medium | 1 | 90 | Medium |
| J07A1075 | 0 | 117 | Ⅱ | + | + | + | 1 | 90 | Medium | 1 | 90 | Medium |
| J07A1076 | 1 | 85 | Ⅱ-Ⅲ | + | - | - | 1 | 90 | Medium | 1 | 90 | Medium |
| J07A1077 | 0 | 116 | Ⅰ | - | - | + | 1 | 70 | Low | 1 | 90 | Medium |
| J07A1079 | 0 | 116 | Ⅰ-Ⅱ | + | + | - | 1.5 | 90 | High | 1 | 90 | Medium |
| J07A1081 | 0 | 115 | Ⅰ-Ⅱ | + | + | - | 1 | 50 | Low | 1 | 90 | Medium |
| J07A1082 | 0 | 115 | Ⅱ | + | + | - | 1 | 80 | Low | 1 | 90 | Medium |
| J07A1084 | 1 | 68 | Ⅱ | - | - | - | 1 | 90 | Medium | 1 | 90 | Medium |
| J07A1085 | 1 | 61 | Ⅱ | + | - | + | 1 | 90 | Medium | 1 | 90 | Medium |
| J07A1087 | 0 | 115 | Ⅱ | - | - | - | 1.5 | 90 | High | 1.5 | 90 | High |
| J07A1088 | 0 | 114 | Ⅱ | - | - | + | 1.5 | 90 | High | 1.5 | 90 | High |
| J07A1089 | 0 | 114 | Ⅰ-Ⅱ | + | + | - | 1 | 90 | Medium | 1 | 90 | Medium |
| J07A1090 | 0 | 114 | Ⅰ-Ⅱ | - | + | + | 1 | 80 | Low | 1 | 90 | Medium |
| J07A1091 | 1 | 113 | Ⅰ-Ⅱ | + | + | + | 1 | 70 | Low | 1 | 90 | Medium |
| J07A1093 | 0 | 113 | Ⅱ | + | - | + | 1 | 90 | Medium | 1 | 90 | Medium |
| J07A1095 | 0 | 112 | Ⅱ | + | - | + | 1 | 90 | Medium | 1.5 | 90 | High |
| J07A1096 | 0 | 112 | Ⅱ | + | + | - | 1 | 90 | Medium | 1 | 90 | Medium |
| J07A1097 | 0 | 112 | Ⅱ | - | - | + | 1 | 70 | Low | 1 | 90 | Medium |
| J07A1098 | 0 | 112 | Ⅱ | - | - | - | 1 | 90 | Medium | 1 | 90 | Medium |
| J07A1099 | 0 | 112 | Ⅱ | + | + | + | 1.5 | 90 | High | 1 | 90 | Medium |
| J07A1100 | 0 | 112 | Ⅱ | + | + | + | 1 | 90 | Medium | 1 | 90 | Medium |
| J07A1101 | 0 | 111 | Ⅰ-Ⅱ/ Ⅱ | + | - | + | 1 | 90 | Medium | 1 | 90 | Medium |
| J07A1102 | 0 | 111 | Ⅱ | + | + | + | 1 | 90 | Medium | 1 | 90 | Medium |
| J07A1103 | 0 | 111 | Ⅱ | + | + | + | 1.5 | 90 | High | 1.5 | 90 | High |
| J07A1105 | 0 | 111 | Ⅱ | - | - | + | 1.5 | 80 | High | 1 | 90 | Medium |
| J07A1106 | 0 | 111 | Ⅱ | + | + | - | 1 | 90 | Medium | 1 | 90 | Medium |
| J07A1107 | 0 | 110 | Ⅱ | + | + | + | 1.5 | 90 | High | 1 | 90 | Medium |
| J07A1109 | 1 | 79 | Ⅱ | + | + | + | 1.5 | 90 | High | 1 | 90 | Medium |
| J07A1110 | 1 | 80 | Ⅱ | + | + | - | 1 | 80 | Low | 1 | 90 | Medium |
| J07A1111 | 0 | 110 | Ⅱ | - | - | - | 1.5 | 90 | High | 1.5 | 90 | High |
| J07A1113 | 0 | 110 | Ⅱ | + | - | - | 1 | 90 | Medium | 1 | 90 | Medium |
| J07A1115 | 1 | 46 | Ⅱ | - | - | - | 1 | 90 | Medium | 1 | 50 | Low |
| J07A1118 | 0 | 109 | Ⅱ | + | + | + | 1 | 90 | Medium | 1 | 10 | Low |
| J07A1120 | 1 | 59 | Ⅱ | + | + | - | 1.5 | 90 | High | 1 | 90 | Medium |
| J07A1121 | 0 | 109 | Ⅱ | - | - | - | 1 | 90 | Medium | 1 | 90 | Medium |
| J07A1122 | 0 | 109 | Ⅱ | - | - | - | 1 | 90 | Medium | 1 | 90 | Medium |
| J07A1124 | 0 | 109 | Ⅱ | + | + | - | 1.5 | 90 | High | 1 | 90 | Medium |
| J07A1125 | 0 | 109 | Ⅱ | - | - | + | 1.5 | 90 | High | 1.5 | 90 | High |
| J07A1126 | 0 | 108 | Ⅱ | + | + | + | 1 | 90 | Medium | 1 | 90 | Medium |
| J07A1127 | 0 | 108 | Ⅱ | - | - | + | 1 | 80 | Low | 1 | 90 | Medium |
| J07A1128 | 0 | 108 | Ⅱ | - | - | - | 1.5 | 90 | High | 1.5 | 90 | High |
| J07A1130 | 1 | 9 | Ⅱ | + | + | + | 1.5 | 90 | High | 1.5 | 90 | High |
| J07A1131 | 0 | 108 | Ⅱ | / | / | / | 1 | 90 | Medium | 1.5 | 90 | High |
| J07A1132 | 0 | 108 | Ⅱ | + | + | + | 1.5 | 90 | High | 1 | 90 | Medium |
| J07A1133 | 0 | 107 | Ⅱ | + | + | - | 1.5 | 90 | High | 1 | 90 | Medium |

State*: 0 is alive; 1 is dead

**Supplementary Materials and Methods**

**Chemicals and antibodies**

DCA, Rotenon and 2-DG were purchased from Sigma-Aldrich. AZD7545 and PGMI-004A were synthesized by Chemfun company (Shanghai, China). Phalloidin was purchased from Cytoskeleton, Inc. The following antibodies were used: PGAM1 (Novus Biologicals); ACTA2 (Abcam); ARP2, ARP3, Cofilin, CAPZA1, Profilin-1, Gelsolin and GAPDH (Cell Signaling); and β-actin (Abmart). Horseradish peroxidase-conjugated anti-mouse and anti-rabbit secondary antibodies were purchased from Kang Chen Bio-tech (Shanghai, China). Alexa Fluor 488 goat anti-rabbit IgG, Alexa Fluor 488 rabbit anti-mouse IgG and Alexa Flour 633 goat anti-rabbit IgG were purchased from Invitrogen.

Cell culture

The human breast adenocarcinoma MDA-MB-231 cell line used in this study was the 4175 subline with high metastatic potential, which was gifted by Dr. Massague (Memorial Sloan-Kettering Cancer Center, New York) and abbreviated as MDA-MB-231 cells. The human breast adenocarcinoma MDA-MB-435, prostate cancer Du145, and human embryonic kidney 293T and HEK293 cells were all purchased from American Type Culture Collection (ATCC). The human lung adenocarcinoma 95D cells were purchased from Cell Resource Center of Shanghai Institutes for Biological Sciences (Shanghai, China), and 293FT cells were purchased from Invitrogen. Cells were cultured according to the supplier’s instructions. Briefly, MDA-MB-231 cells were grown in Dulbecco’s modified Eagle medium (DMEM; Gibco) supplemented with 1 mM sodium pyruvate and 10% FBS (Gibco); MDA-MB-435 293FT, 293T, and HEK293 cells were cultured in DMEM supplemented with 10% FBS. 95D and Du145 cells were grown in RPMI-1640 medium (Gibco) supplemented with 10% FBS. Cells were grown in humidified atmosphere of 5% CO_2_ at 37°C.

Plasmids

Wild-type PGAM1 plasmid was purchased from Addgene and subcloned into the pcDNA3.1 (-)-flag vector (Addgene) at the BamHl and Xholl restriction sites. N160 (1-160 aa), N200 (1-200 aa), N220 (1-220 aa), N235 (1-235 aa) and N250 (1-250 aa) PGAM1 truncations, and Δ201-220 (201-220 aa deleted), Δ201-205 (201-205 aa deleted), Δ206-210 (206-210 aa deleted) and Δ211-215 (211-215 aa deleted) PGAM1 deletions were constructed using recombinant polymerase chain reaction. H11R, R62K, H186R PGAM1 mutants were generated using the Muta-direct Site-directed Mutagenesis kit (Beijing SBS Genentech Co, Ltd, Beijing, China). The template sequences for PGAM1 short hairpin RNA (shRNA) and non-silencing shRNA (Scr) obtained using the Insert Design Tool for the pLKO.1/Puro vector (Addgene) were subcloned into pLKO.1/Puro vector (Addgene). All the constructs were sequenced by Invitrogen Company (Shanghai, China).

**siRNA and plasmids transfection**

For siRNA transfection, cells were plated at 30%-60% confluence in OPTI-MEM serum-free medium and transfected with a specific siRNA duplex using Lipofectamine RNAiMAX Reagent Agent (Life Technologies) according to the manufacturer’s instructions. siRNAs against ACTA2 were siACTA2 pool purchased from Santa Cruz. The rest siRNAs were ordered as RP-HPLC-purified duplexes from Ruibo (Guangzhou, China). Target sequences for siRNAs were as follows: siPGAM1-1: 5’-CGA CTG GTA TTC CCA TTG TTT-3’; siPGAM1-2: 5’-GTC CTG TCC AAG TGT ATC TTT-3’; siPGAM1-3: 5’-GGA AAC GTG TAC TGA TTG CTT-3; siHK2: 5’-CCG TAA CAT TCT CAT CGA TTT-3’; siPKM2: 5’-GAG GCT TCT TAT AAG TGT TTA-3’; siPDHK1: 5’-AGT CGC ATT TCA ATT AGA A-3’; siLDHA: 5’-CAG TAT CTT AAT GAA GGA CTT-3’; non-targeting control siRNA (NC): 5’-ATC TTA ATG AAG GAC TT-3’.

For plasmid transfections, cells were grown to 60% confluence in 6-cm dishes and transfected with 4 μg of plasmids using 4 μL of Lipofectamine 2000 (Invitrogen) according to the manufacturer’s instructions. For all experiments using PGAM1 truncations and deletions (N160, N200, Δ201-220, Δ201-205, Δ206-210, Δ211-215), MG-132 at 10 µM was added at 6 hr prior to the harvest of the cells.

**shRNA and infection**

Targeting sequences for the shRNAs were as follows: shPGAM1-1: 5’-CCA TCC TTT CTA CAG CAA CAT-3’; shPGAM1-2: 5’-CCT GTG AGA GTC TGA AGG ATA-3’; shPGAM1-3: 5’-CGC CTC AAT GAG CGG CAC TAT-3’; Scramble (Scr): 5'-CCG GCA AAT CAC AGA ATC GTC GTA TCT CGA GAT ACG ACG ATT CTG TGA TTT GTT TTT G-3'.

293FT cells were grown in DMEM supplemented with 10% FBS to 60% confluence. The transfection complex was prepared as follows according to the manufacturer’s instructions: the pLKO.1-PGAM1 shRNA vector was added to Lipofectamine2000 (Invitrogen) in 500 μl OPTI-MEM serum-free medium along with pCMV-dR8.2 dvpr packaging vector and pCMV-VSV-G envelop vector (4:3:1). About 6 hr after transfection, the media was replaced by fresh DMEM containing 10% FBS. After additional 48-hr incubation at 37°C, the supernatant was collected, filtered with a 0.45-μm filter (Millipore), and infected MDA-MB-231 cells in the presence of 8 μg mL^-1^ polybrene (Millipore) for 24 hr. Cells stably expressing PGAM1 shRNA were selected in the presence of puromycin (1 μg ml^-1^) before plating for experiments.

Cell proliferation assay

Cells were seeded into 96-well plate and cell proliferation was assessed using CCK8 assay (Life Technologies). The absorbance (optical density, OD) was read at a wave length of 450 nm on an ELISA plate reader.

Transwell assay

Cells were starved in serum-free and growth factor-free medium for 12 hr and added to the top chambers of 24-well Transwell plates (8 μm; Corning Costar Corp). The bottom chambers were filled with growth medium supplemented with 15% FBS. After incubation for 6 or 12 hr at 37°C, non-migrating cells were removed from the upper chamber with a cotton swap. Migrated cells were fixed in paraformaldehyde (4%) and stained with crystal violet solution for 15 min at room temperature. Each well was photographed under a light microscope at a magnification of 100 x. Motility was quantified by addition of 100 μL 33% acetic acid, and the absorbance of the resulting solution was measured at 570 nm using a multiwell spectrophotometer (VERSAmax; Molecular Devices, Sunnyvale, CA). In parallel, cell proliferation during a 12-hr culture was measured using CCK-8 assay by measuring absorbance at 450 nm. The assay was performed at least three independent times. Quantification of cell migration was normalized by the cell numbers to exclude the impact of cell growth.

**Wound-healing assay**

Cells were grown to confluence and the wound was induced by scraping the cell monolayer with a pipette tip p/200 and left for 16 hr before photographing. After scraping the wound, the growth medium were replaced with the one supplemented with 3% FBS. Each wound was photographed under a light microscope at a magnification of 100x at 0 hr and 16 hr. The assay was performed at least three independent times. Quantification of cell migration was performed by quantifying the total distance of the wound.

Cell track assay

Incucyte ZOOM determined cell track assay was used to detect cancer cell motility. Cells were seeded into 6-well plate. After attachment, Incucyte ZOOM was used to take photos of cancer cell morphology every one hour for 24 hr. The tracks of cancer cells were analyzed by software ImageJ (MtrackJ), and the velocity of cancer cells were calculated. Each experimental well and control well were selected in 3 different fields of vision in cancer cells for analysis. The assay was performed at least three independent times.

Co-immunoprecipitation

Total cell lysates were prepared by incubating cells with NP-40 lysis buffer (20 mM Tris-HCl pH 8.0, 150 mM NaCl, 1% Nonidet P40, and 2 mmol L-1 EDTA) supplemented with a protease inhibitor cocktail (Roche) on ice for 30 min. Lysates were centrifuged at 14,000 g for 15 min. Protein concentration of the supernatants was measured using BCA protein assay kit (Beyotime). Anti-flag M2 beads (Sigma; A2220) were incubated with the supernatants for 2 hr at 4°C. To test the interaction between endogenous PGAM1 and ACTA2, anti-ACTA2 antibody (Abcam; ab7817) was incubated with the supernatants for 4 hr at 4°C, and then added the Protein A/G PLUS-Agarose (Santa Cruz; sc-2003) to incubate 2 more hr at 4°C. The beads were collected and washed six times with cold lysis buffer NP40. The bound proteins were eluted by boiling with 1x loading buffer containing 0.2% sodium dodecyl sulfate and 100 mM DTT. The supernatant was collected and separated using SDS-PAGE.

Immunoblotting

Cells were lysed using preheated 2% SDS by vortexing vigorously for 2-3 sec followed by boiling for 30 min. Protein concentrations were determined using a BCA assay (Thermo Scientific). Proteins were subjected to SDS-PAGE separation and transferred to PVDF membranes (Immobilon-P, Millipore). Membranes were blocked for 1 h at room temperature with 3% milk in 1× Tris-buffered saline Tween-20 (TBST) (25 mM Tris, 150 mM NaCl, 2 mM KCl, pH 7.4, supplemented with 0.2% Tween-20) and blotted with primary antibodies at 4°C overnight. After washing with TBST three times for 30 min, membranes was incubated with horseradish peroxidase-conjugated anti-rabbit IgG (dilution, 1:2000) or anti-mouse IgG (dilution, 1:2000) antibodies at room temperature for 1 h. The membranes were washed with TBST for three times before visualized using Western Blotting Luminal Reagent (Pierce) and subsequent exposure to KODAK X-OMAT BT Film (Kodak, Rochester, NY). All experiments were performed at least three independent times.

**Cellular F-actin and G-actin**

Cells were starved in serum- and growth factor-free medium for 12 hr and then stimulated with growth medium supplemented with 15% FBS for 1 hr before harvesting. Cellular F-actin and G-actin level was examined using G-Actin/F-actin In Vivo Assay Biochem Kit (BK037, Cytoskeleton) following the manufacturer’s instructions.

**Fluorescence microscopy**

Cells grown on coverslips were fixed in 4% paraformaldehyde in PBS for 10 min at 37°C, rinsed three times with PBS, and permeabilized with PBS containing 0.1% Triton X-100 for 30 min at room temperature. After coverslips were rinsed three times with PBS, cells were blocked with 5% bovine serum albumin (TBST containing 5% bovine serum albumin) at room temperature for 15 min and incubated with primary antibodies or Texas red-conjugated phalloidin (4 U ml^-1^; Molecular Probes, Eugene, OR, USA) for 1 hr at room temperature. Afterwards, cells were washed three times with TBST and incubated for 30 min at room with Alexa-Fluo 633-labeled goat anti-mouse or Alexa-Fluor 488-labelled goat anti-rabbit IgG. The slides were mounted on VECTASHIELD mounting medium (Vector Laboratories, Burlingame, CA) and images were captured by confocal microscopy using a Leica DMRB (Leica, Solms, Germany).

**Oxygen consumption rate and extracellular acidification rate analysis**

Cells were planted into XF96 cell culture plates (Seahorse Bioscience). Each XF96 assay well was equipped with a disposable sensor cartridge and embedded with 96 pairs of fluorescent biosensors (oxygen and PH), coupled to fiber-optic waveguides. The measurement of oxygen consumption was expressed in pmol min^-1^ and extracellular acidification rate was expressed in mPH min^-1^.

**Measurement of intracellular PHGDH and 6-PGD activity**

Intracelluar activity of PHGDH and 6-PGD was measured according to previously described[^1^](#_ENREF_1). In details, cells were grown on 6-well plate and lysed using 50 μL NP-40 lysis buffer (20 mM Tris-HCl pH 8.0, 150 mM NaCl, 1% Nonidet P40, and 2 mM EDTA) supplemented with protease inhibitor cocktail (Roche). PHGDH activity was measured by the NADH production rate. The reaction buffer containing 200 mM Tris-HCl (pH8.1), 400 mM KCl, 0.6 mM NAD, 2 mM GSH, 10 mM EDTA was mixed with cell lysates. The reaction was initiated by the addition of 3-PG to a final concentration of 10 mM and followed by measuring the absorbance at 340 nm over a 10 min period. 6-PGD activity was determined by the NADPH production rate. Reaction Substrate concentrations were as follows: 0.2 mM 6-phosphogluconate, and 0.1 mM NADP+. The lysates were added to a cuvette containing buffer (50 mM Tris, 1 mM MgCl2, pH 8.1) and then the reaction was initiated by NADP+ and followed by measuring the absorbance at 340 nm. The absorbance (optical density, OD) was measured by a DU800 spectrophotometer (Beckman).

**Experimental lung metastasis study**

Female Balb/c nude mice at 6 weeks of age were housed five or six mice per cage in a specific pathogen-free room with a 12-hour light/dark schedule at 25°C ± 1°C and were fed an autoclaved chow diet and water ad libitum. All experiments were performed in strict accordance with the Guide for the Care and Use of Laboratory Animals of the National Institutes of Health. The mice were randomly divided into two groups before the injection. 1 × 10^6^ MDA-MB-231-Scramble cells or MDA-MB-231-shPGAM1-1 cells resuspended in 100 µL PBS were injected into the lateral tail veins of mice (n = 15 per group). After 18 days, the mice were killed by CO_2_, their lungs were removed and fixed with paraformaldehyde (4%) before dehydration and paraffin embedding. Paraffin sections from lung big lobe were stained with hematoxylin and eosin (H&E) according to standard protocols or were subjected to immunohistochemical staining using a horseradish peroxidase-labeled streptavidin-biotin ABC kit (ZSGBBIO, Beijing, China) with hematoxylin as the counterstain (two independent experiments). Anti-PGAM1 was diluted 1:100 in 0.1% BSA/PBS and incubated on slides in a humidified chamber for 2 hr. 20 serial lung section at metastasis nodules enriched lung area from each mice were histologically analyzed and were imaged at ×100 magnification. Metastatic foci was defined as area of consolidation in lung parenchyma composed of apparent heterogeneous cell populations of irregular cancer cell alignment and nuclear hyperchromatism. The quantification of metastatic foci in each section was performed by double-blinded counting. 3 out of 20 sections per mice were randomly picked for quantification.

# Tissue microarray analysis

The breast tumor tissue microarrays containing 160 samples of invasive ductal carcinoma and immunohistochemistry staining were conducted by Shanghai Outdo Biotech Company. The expression of PGAM1 and ACTA2 were determined using a set of criteria by two independent pathologists blinded to the clinicopathologic condition. Briefly, staining intensity in the cytoplasm was graded using a scale from 0 to 3 (0 for no immunostaining, 1 for light-brown color, 2 for medium-brown color, and 3 for dark-brown color). The percentage of positively stained cells was scored as follows: 0, no staining; 1, <25 % of the entire malignant cell population; 2, 25-75% of the entire malignant cell population; 3, >75 % of the entire malignant cell population. The final composite score was the product of the intensity multiplied by and percentage scores, being classified as: high, medium and low subgroups. Those with a score ≥ 100 were defined as high expression (n = 48, 30%), score ≤80 were considered as low expression (n = 63, 39%), and score between 80 and 100 were subgrouped as medium (n = 49, 31%). Discrepancies in scoring were resolved through discussion.

**Supplementary References**

1. Hitosugi T, et al. Phosphoglycerate mutase 1 coordinates glycolysis and biosynthesis to promote tumor growth. Cancer Cell 22, 585-600 (2012).
